# PET-based radiomics visualizes tumor-infiltrating CD8 T cell exhaustion to optimize radiotherapy/immunotherapy combination in mouse models of lung cancer

**DOI:** 10.1186/s40364-023-00454-z

**Published:** 2023-01-25

**Authors:** Ying Zhang, Hui-Hui Hu, Shi-Hong Zhou, Wu-Yan Xia, Yan Zhang, Jian-Ping Zhang, Xiao-Long Fu, Wen Yu

**Affiliations:** 1 Department of Radiation Oncology, Shanghai Chest Hospital, Shanghai Jiao Tong University, 241 West Huaihai Road, Shanghai, 200030 China; 2 Department of Thoracic Surgery, Shanghai Chest Hospital, Shanghai Jiao Tong University, Shanghai, China; 3 Shanghai Institute of Immunology, Department of Immunology and Microbiology, State Key Laboratory of Oncogenes and Related Genes, Shanghai Jiao Tong University School of Medicine, Shanghai, China; 4 Department of Nuclear Medicine, Fudan University Shanghai Cancer Center, Shanghai, China

**Keywords:** Radiotherapy, Immunotherapy, Tumor microenvironment, CD8^+^ T cell exhaustion, Radiomics

## Abstract

**Background:**

Cumulative preclinical and clinical evidences showed radiotherapy might augment systemic antitumoral responses to immunotherapy for metastatic non-small cell lung cancer, but the optimal timing of combination is still unclear. The overall infiltration and exhausted subpopulations of tumor-infiltrating CD8^+^ T cells might be a potential biomarker indicating the response to immune checkpoint inhibitors (ICI), the alteration of which is previously uncharacterized during peri-irradiation period, while dynamic monitoring is unavailable via repeated biopsies in clinical practice.

**Methods:**

Basing on tumor-bearing mice model, we investigated the dynamics of overall infiltration and exhausted subpopulations of CD8^+^ T cells after ablative irradiation. With the understanding of distinct metabolic characteristics accompanied with T cell exhaustion, we developed a PET radiomics approach to identify and visualize T cell exhaustion status.

**Results:**

CD8^+^ T cell infiltration increased from 3 to 14 days after ablative irradiation while terminally exhausted populations significantly predominated CD8^+^ T cells during late course of this infiltrating period, indicating that 3–7 days post-irradiation might be a potential appropriate window for delivering ICI treatment. A PET radiomics approach was established to differentiate T cell exhaustion status, which fitted well in both ICI and irradiation settings. We also visualized the underlying association of more heterogeneous texture on PET images with progressed T cell exhaustion.

**Conclusions:**

We proposed a non-invasive imaging predictor which accurately assessed heterogeneous T cell exhaustion status relevant to ICI treatment and irradiation, and might serve as a promising solution to timely estimate immune-responsiveness of tumor microenvironment and the optimal timing of combined therapy.

**Supplementary Information:**

The online version contains supplementary material available at 10.1186/s40364-023-00454-z.

## Introduction

Combination of radiotherapy (RT) and immune checkpoint inhibitors (ICI) therapy has demonstrated its great potential to improve the treatment outcome of patients with advanced non-small cell lung cancer (NSCLC) [1–4]. While the optimal timing and pattern of combination treatments is still unclear, and reliable biomarkers to select patients who would benefit most from the RT/ICI combination treatments are urgently needed [5].

The infiltration abundance of tumor-infiltrating lymphocytes (TILs), especially CD8^+^ T cells, in tumor microenvironment (TME) has been found to be a potential biomarker of response to ICI or prognosis [6–9]. Evidence showed that the infiltration density of CD8^+^ TILs may experience alteration during peri-irradiation period, which is closely correlated with the prognosis and pathologic response after chemoradiotherapy [10]. Therefore, biomarkers based on baseline level or few time points may be insufficient to provide precise guidance to clinical strategies.

Moreover, as antigens persist in tumors, CD8^+^ T cells may differentiate into a special state known as T cell exhaustion [11]. Exhausted CD8^+^ T cells (Tex) exhibit progressive loss of effector functions and high expression of inhibitory receptors, such as PD-1, TIM-3 and so on [12, 13], which dampen effector immunity and cause poor responsiveness to ICI therapy. Early-exhausted T cells (early Tex) with intermediate expression of PD-1(PD-1^int^) can be reinvigorated by blockade of the PD-1 pathway whereas terminally-exhausted T cells (terminal Tex) with high expression of PD-1(PD-1^hi^) cannot [12]. Recent evidence indicates that the proportion of early Tex versus terminal Tex was associated with the prognosis of patients with NSCLC, and might allow to identify patients who may benefit from PD-1 blockade or combined therapies [14] [15]. Therefore, dynamic monitoring of both the overall infiltration abundance and the proportion of different functional subpopulations of CD8^+^ T cells during peri-irradiation period might be a promising and efficient approach to indicate the ICI responders as well as the timing of combining ICI therapy with RT.

However, the invasiveness of repeated biopsies and sampling bias due to the heterogeneity of tumors bring great difficulties to acquire dynamic biomarkers based on histopathological level. Radiomics, a noninvasive “digital biopsy” technique, which extracts quantitative features from medical images to correlate with biological characteristics of tumors, might provide a solution [16]. Radiomics approaches have been developed to evaluate the prognosis in various tumors [17–21], and even to predict tumor-infiltrating CD8^+^ cells, response to ICI therapy and abscopal response of combining ICI therapy with RT [22, 23]. Since exhausted T cells have been reported to exhibit metabolic insufficiency with suppressed glycolysis and restricted glucose uptake [24, 25], we assumed that metabolic characteristics of Tex might be reflected by radiomic features of ^18^FDG-PET.

Here, basing on tumor-bearing mice model, we aimed to investigate the dynamics of overall infiltration and functional subpopulations of CD8^+^ T cells after ablative irradiation, and to develop a PET radiomic approach to identify and visualize the level of T cell exhaustion.

## Methods and materials

### Cell lines

The murine Lewis lung carcinoma (LLC) cells and B16 melanoma cells were purchased from the National Collection of Authenticated Cell Cultures (Shanghai, China). Cells were cultured in RPMI Medium 1640 (Gibco), which contained 10% fetal bovine serum (Gibco) and 1% penicillin/streptomycin (Hyclone). Cells were incubated at 37℃ in 5% CO_2_.

### Tumor models and treatments

Female C57BL/6 N mice between six to eight weeks old were purchased from Zhejiang Vital River Laboratory Animal Technology Co. Ltd (Jiaxing, Zhejiang, China) and were maintained under specific pathogen-free (SPF) conditions, with the temperature of 20 ~ 25℃, humidity of 40% ~ 70%, and the light time set from 8:00 to 20:00.

To establish tumor models, 1 M LLC or 0.5 M B16 cells were inoculated subcutaneously into the right flank of mice. Treatment was started when average tumor volume reached 200mm^3^ (10 days after inoculation, defined as D0). Mice were randomized to receive irradiation at D0 (RT group) or 200 μg anti-PD-L1(10F.9G2, BioXcell) antibody i.p. since D7, every 3 days for 5 times (ICI group). Three mice per group per interested time point were used in each of the three independent experiments. All mice experiments were ethically approved by the Institutional Animal Care and Use Committee of Shanghai Chest Hospital.

### Irradiation

Mice were anesthetized and positioned in the prone orientation. A CT scan was acquired and treatment planning was performed using Monte Carlo algorithm. Dose-volume histogram evaluation showed the target volume receiving 95% ~ 105% of the dose. Radiation was delivered using a 6MV X-ray linear accelerator at a dose rate of 600 cGy/min.

### Micro-PET/CT

For RT group, micro-PET/CT was performed on D0, D3, D7, D14 and D21. While for ICI group, micro-PET/CT was performed on D0, D7 (before anti-PD-L1 treatment) and D22 (after anti-PD-L1 treatment). Mice were injected intraperitoneally with 5.55 MBq ^18^F-FDG in 0.1 mL saline. After an hour, micro-PET/CT images were acquired using an Inveon micro-PET/CT scanner (Siemens Preclinical Solution). During scanning period, mice were maintained under 2% isoflurane anesthesia and were placed in the prone position on the bed of the scanner. CT scans were performed first (Voltage: 80 kV, Current: 500μA, Exposure 300 ms, Projections: 120, effective pixel size: 112.85 μm). Then, PET scans were performed 10 min later, OSEM3D/SP-MAP algorithm (2 OSEM iterations, 18 MAP iterations, target resolution 1.5 mm and matrix size: 128 × 128) without filtering and smoothing was used to reconstruct PET images. The PET images were converted into SUV units by normalizing the activity concentration to the dosage of injected ^18^F-FDG and mouse body weight. The micro-PET and CT images were generated separately and then fused using Inveon Research Workplace (Siemens Preclinical Solution).

### RNA extraction and microarray analysis

RNA was collected from LLC tumors either before irradiation (D0) or at day 3, 7, 14, 21 post-irradiation (D3, D7, D14 and D21). Total RNA was quantified by the NanoDrop ND-2000 (Thermo Scientific) and the RNA integrity was assessed using Bioanalyzer 2100 (Agilent). The sample labeling, microarray hybridization and washing were performed based on the manufacturer's standard protocols. In brief, total RNA was transcribed to double strand cDNA, then synthesized into cRNA and labeled with Cyanine-3-CTP. The labeled cRNAs were hybridized onto the microarray. After washing, the arrays were scanned by the Agilent Scanner G2505C (Agilent). The Agilent Mouse Gene Expression 4*44 K v2 Microarray (DesignID: 026,655) was used in this experiment and data analysis was conducted by OE Biotechnology Co. Ltd (Shanghai, China).

### Cibersort analysis

The abundance of tumor-infiltrating immune cell subsets was estimated by a deconvolution approach with online analytical tool CIBERSORTx (Cell type identification by estimating relative subsets of known RNA transcripts; https://cibersortx.stanford.edu) [26, 27]. The CIBERSORT LM22 matrix, consisting of 547 genes, transforms gene-expression data of microarray analysis into relative fractions of immune cells phenotypes. CIBERSORT implements Monte Carlo sampling to generate an empirical P-value for the deconvolution. Only cases with a *P*-value < 0.05, which indicated a reliable estimation of immune cell infiltration, were used for further analysis.

### Flow cytometry

Tumor samples were disposed with Tumor Dissociation Kit (Miltenyi Biotec). Then single-cell suspensions from tumors were lysed to remove red blood cells using RBC Lysis/Fixtion Solution (BioLegend) and filtered through a 70 μm MACS SmartStrainer (Miltenyi Biotec). After that, the single-cell suspensions were incubated with fluorescently labeled antibodies (BioLegend) against CD45, CD3, CD4, CD8 and PD-1 at 4℃ for 45 min. For intracellular staining, cells were fixed and permeabilized, and then incubated with TIM-3 (BioLegend), TOX (eBioscience), TCF-1 (BD) at 4℃ for 6 h. Multicolor flow cytometry analysis was conducted with BD Fortessa flow cytometer. Expression level of PD-1 on CD8^+^ T cells was gated by its low controls of CD8^+^ T cells from spleen of untreated mice (PD-1^low^), high controls of cells stained by both anti-TOX and anti-PD-1(PD-1^hi^) and between them (PD-1^int^) [15] (Details are provided in the Supplementary Methods section). The proportion of early Tex (CD8^+^PD-1^int^) and terminal Tex (CD8^+^PD-1^hi^) in CD8^+^ T cells, the ratio of early to terminal Tex (E/T), and mean fluorescence intensity (MFI) of PD-1 expression on CD8^+^ T cells were further analyzed using FlowJo software (Treestar).

### Radiomics analysis

Tumors were manually delineated on PET/CT images using MIM Maestro Version 7.1.4 (MIM Software), excluding adjacent organs, bone structure and large vessels. Radiomics features were extracted with Imaging biomarker explorer (IBEX) [28], 57 from CT images and 61 from PET images, including first-order features, shape features, and textural features derived from Gray Level Cooccurence Matrix (GLCM) and Gray Level Run Length Matrix (GLRLM) (Table S1). Radiomics features would undergo a two-step selection. First, the feature reproducibility under varying scanning conditions was assessed by test–retest analysis [29, 30], using RIDER Lung CT and RIDER Phantom PET-CT datasets from The Cancer Imaging Archive (TCIA). Concordance correlation coefficient (CCC) was calculated and features with good robustness would be selected for further analysis. Second, with the ICI group as the training set, least absolute shrinkage and selection operator (LASSO) logistic regression was used to select radiomic features significantly correlated with characteristics of T cell exhaustion, such as the infiltrating proportion of early Tex and terminal Tex among CD8^+^ T cells, E/T, or MFI of PD-1 on CD8^+^ T cells. Then, significant features were further collected to establish a binary logistic regression model for identifying the level of T cell exhaustion (median proportion of terminal Tex as cutoff). Finally, validation of the model was performed with RT group as external validation set. Concordance index (C-index) and calibration plot were used to evaluate the discriminability and calibration of this model. The goodness-of-fit of model was assessed by Brier score.

### Statistical analysis

Data are presented as the mean $\pm$ SEM. For comparing different time groups, one-way analysis of variance with Bonferroni corrections was applied with two-sided *P*-values (^#^*P* < 0.1, *P < 0.05, **P < 0.01, ***P < 0.001). Statistical analyses were performed using R software 4.1.2 and GraphPad Prism 8.4.3.

### Data availability

The data generated in this study are available within the article and its supplementary data files.

## Results

### CD8^+^ T cell infiltration increased from 3 to 14 days after ablative irradiation

**Fig. 1:**
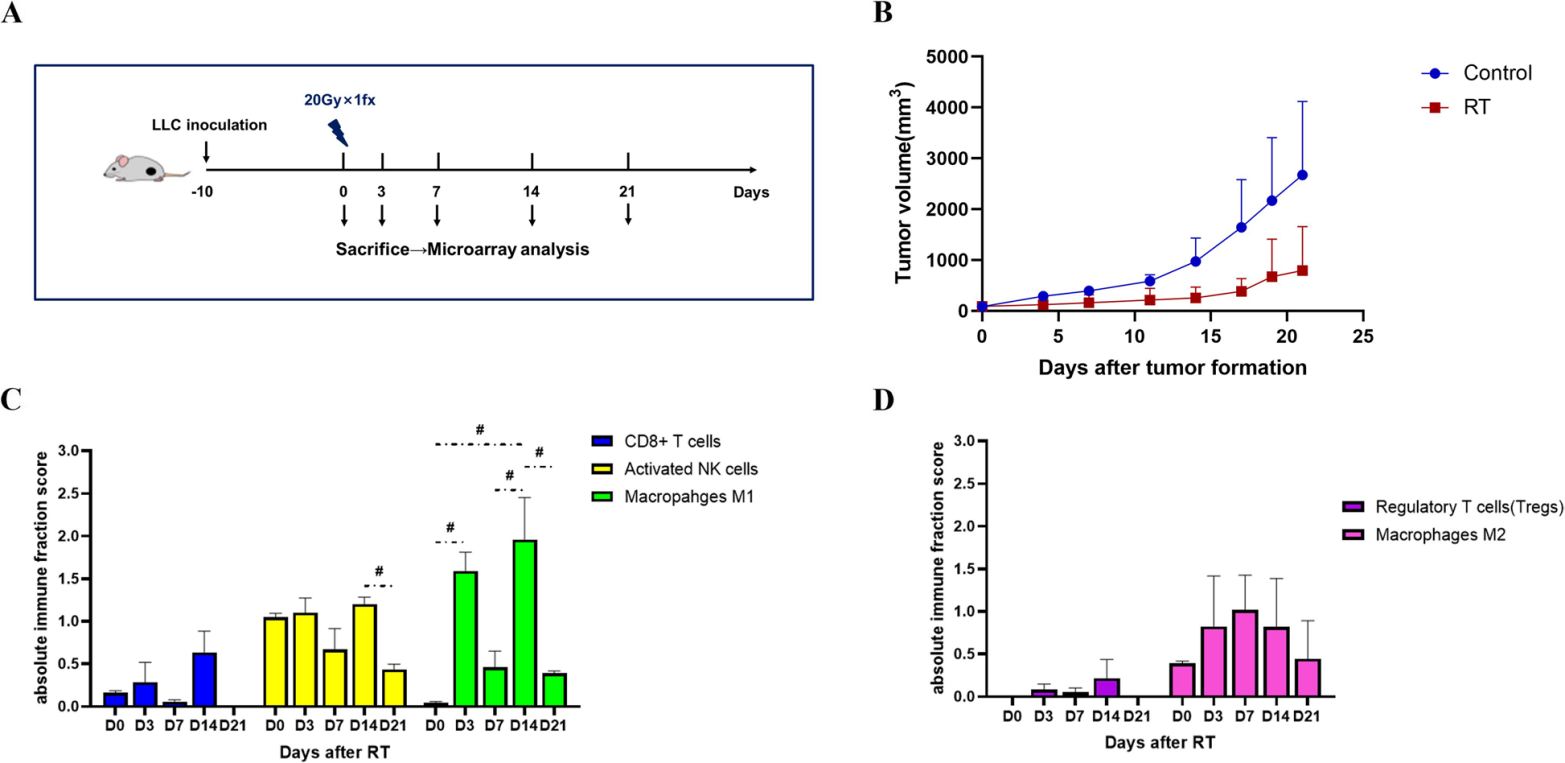
Ablative irradiation induced a hot tumor microenvironment within 2 weeks. **A** Experiment scheme. **B** Tumor growth of LLC model in RT group and control group. **C** and **D** The infiltration of immune-promoting cells (**C**) and immunosuppressive cells (**D**) in LLC tumor microenvironment over the time after ablative irradiation. The absolute immune fraction score was produced by CIBERSORT to quantitatively measure the overall abundance of each cell type. #: *P* < 0.1

To assess the immune cell infiltration abundance, we collected LLC tumors at D0, D3, D7, D14 and D21 after ablative irradiation with a single fraction of 20 Gy (Fig. 1A, B) and used CIBERSORT to profile overall immune content in microarray datasets. It was showed that, after ablative irradiation, the tumor-infiltrating CD8^+^ T cells increased from D3 through the peak at D14, and then declined sharply to a below-baseline level at D21. Immune-promoting cells of innate immune system, such as activated NK cells and M1 macrophages, also exhibited an increasing trend similar to that of CD8^+^ T cells, peaking at D14 (Fig. 1C). However, the infiltration of immunosuppressive cells, including regulatory T cells (Treg) and M2 macrophages, varied slightly after ablative irradiation (Fig. 1D). Hence, ablative irradiation to the tumor induced a “hot” microenvironment within 2 weeks, with both innate and adaptive immune systems activated, whereas after that it was tending towards a “cold” microenvironment.

### Anti-PD-L1 antibody reinvigorated CD8^+^ T cell exhaustion in different degrees

**Fig. 2:**
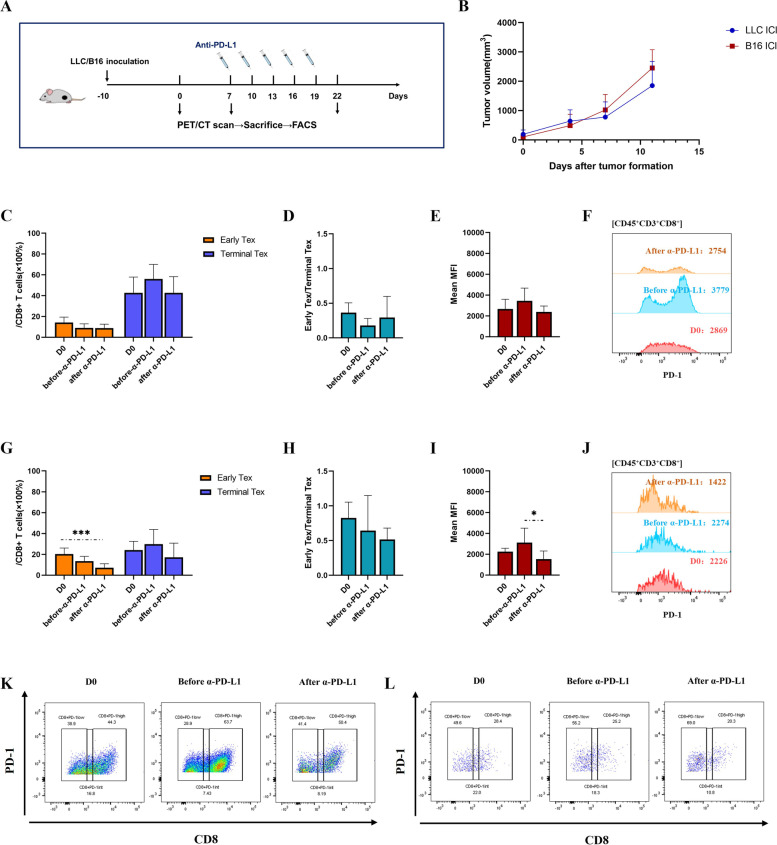
Anti-PD-L1 antibody reinvigorated CD8^+^ T cell exhaustion in different degrees. Flow cytometric analysis of CD8^+^ T cell exhaustion before and after anti-PD-L1 intervention in LLC and B16 models. **A** Experiment scheme. **B** Tumor growth of LLC model and B16 model in ICI group. **C** The proportion of early and terminal Tex cells in CD8^+^ T cells of LLC model in ICI group. **D** The ratio of early-exhausted CD8^+^ T cells to terminally-exhausted CD8^+^ T cells (E/T) of LLC model in ICI group. **E** and **F** Mean Fluorescence intensity (MFI) of PD-1 expression in CD8^+^ T cells of LLC model. The data are presented as summary graph (**E**) and representative plot (**F**). **G** The proportion of early and terminal Tex cells in CD8^+^ T cells of B16 model in ICI group. **H** E/T of B16 model in ICI group. **I** and **J** MFI of PD-1 expression in CD8^+^ T cells of B16 model. The data are presented as summary graph (**I**) and representative plot (**J**). **K** and **L** Representative flow cytometric staining of exhausted CD8^+^ T cells subsets of LLC (**K**) and B16 models (**L**). The data shown are representative of three independent experiments (*n* = 3 per time point) and are presented as the mean ± SEM. *: *P* < 0.05, ***: *P* < 0.001

To evaluate the effect of ICI treatment on CD8^+^ T cell exhaustion and to induce heterogeneous exhaustion status, we administrated five-dose anti-PD-L1 antibody to LLC-mice model (Fig. 2A, B) and performed flow cytometry to capture tumor-infiltrating CD8^+^ T cells of different exhaustion status (Fig. S1). As comparison to the ICI-resistant LLC model, the same experiment was performed on ICI-responsive B16 model [31]. With a baseline high level of T cell exhaustion (the proportion of terminal Tex in CD8^+^ T cells ~ 40%), the proportion of early Tex of LLC model did not decrease obviously after anti-PD-L1 intervention, neither did mean MFI of PD-1 expression on CD8^+^ T cells (Fig. 2C, D, E, F and K). In contrast, B16 model initially possessed a less exhausted T cell population (the baseline proportion of terminal Tex in CD8^+^ T cells ~ 20%), and the proportion of early Tex as well as mean MFI of PD-1 expression of CD8^+^ T cells significantly declined after anti-PD-L1 intervention, suggesting a remarkable reinvigoration of T cell exhaustion (Fig. 2G, H, I, J and L). This result is consistent with other previous researches, which have discovered that the exhaustion of tumor-infiltrating CD8^+^ T cells can be reinvigorated by anti-PD-L1 antibody [32, 33].

### CD8^+^ T cell exhaustion peaked at day 7 through 14 after ablative irradiation

**Fig. 3:**
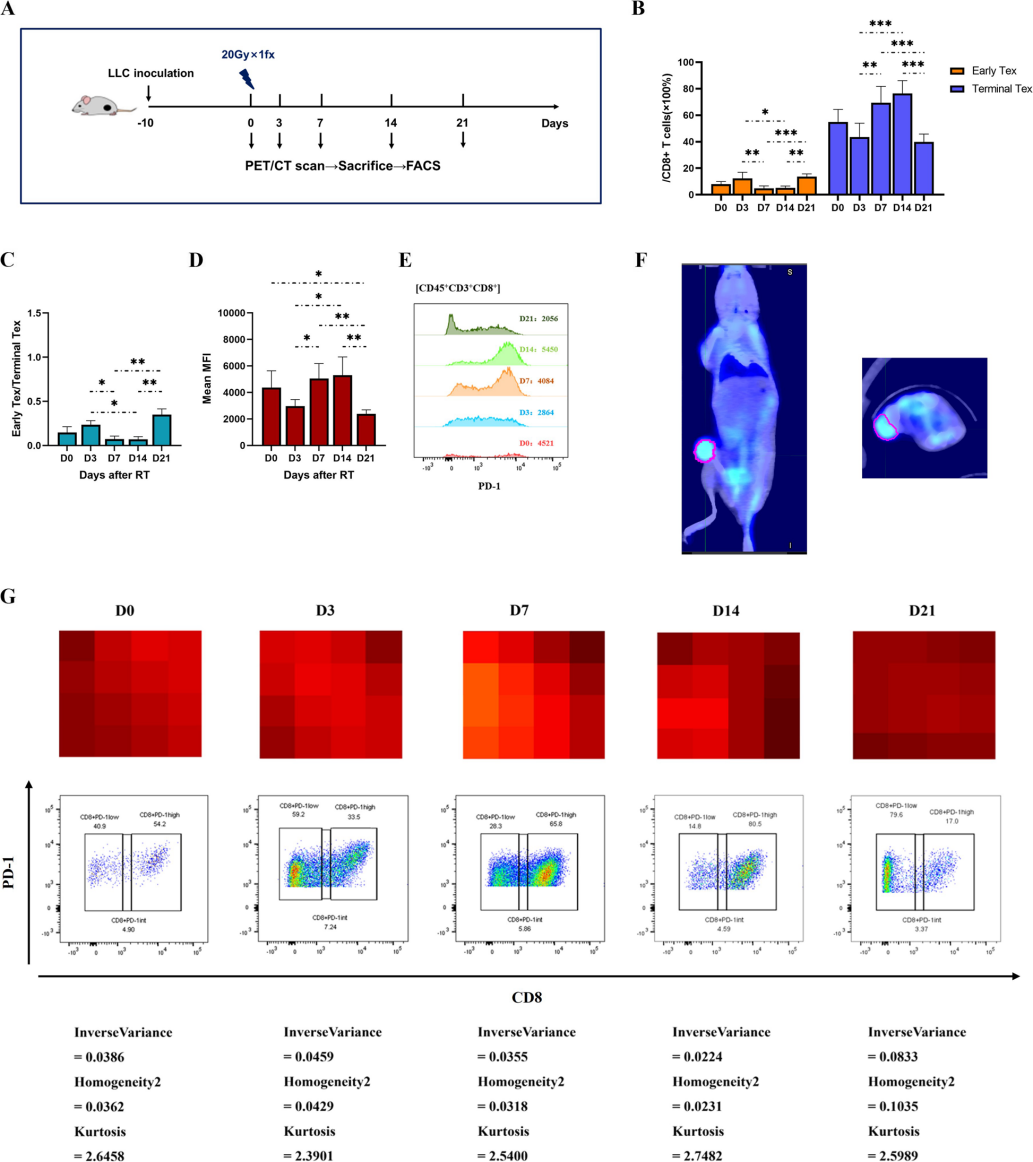
CD8^+^ T cell exhaustion peaked at day 7–14 after ablative irradiation and could be visualized by PET radiomics features. Flow cytometric analysis of CD8^+^ T cell exhaustion after ablative irradiation in LLC model. **A** Experiment scheme. **B** The proportion of early and terminal Tex cells in CD8^+^ T cells. **C** The ratio of early-exhausted CD8^+^ T cells to terminally-exhausted CD8^+^ T cells (E/T). **D** and **E** Mean Fluorescence Intensity (MFI) of PD-1 expression in CD8^+^ T cells. The data are presented as summary graph (**D**) and representative plot (**E**). **F** Representative coronal and cross-sectional PET/CT images for a tumor-bearing mouse. Region of interest (ROI) was manually delineated to cover the entire tumor on fusion images, excluding adjacent organs, bone structure and large vessels (pink line). Radiomics features would be extracted from ROI for further analysis. **G** Local homogeneity on PET images was negatively correlated with the proportion of terminal Tex cells (CD 8^+^PD-1^hi^) in CD8^+^ T cells. Representative PET pixel images of xenografts (upper row, brighter pixels with higher ^18^FDG uptake) and corresponding flow cytometric plots (middle row) are showed at each time point, along with radiomics metrics (lower row). The data shown are representative of three independent experiments (*n* = 3 per time point) and are presented as the mean ± SEM. *P < 0.05, ** *P* < 0.01, *** *P* < 0.001

To elucidate the effect of ablative irradiation on CD8^+^ T cell exhaustion, flow cytometry was further performed at a serial of time points after LLC tumors received a single fraction of 20 Gy (Fig. 3A). The terminal Tex proportion did not change significantly during the first 3 days after irradiation, but then quickly increased and reached the peak at day 7 through 14 (69.44% ~ 76.35%), and sharply dropped to a below-baseline level after that (Fig. 3B, C, and middle row of G). Similar trend was observed in mean MFI of PD-1 expression on CD8^+^ T cells (Fig. 3D, E).

### Local homogeneity on PET images was negatively correlated with the level of CD8^+^ T cell exhaustion

To further explore the PET/CT radiomics features which could non-invasively identify CD8^+^ T cell exhaustion levels, radiomics analysis was performed (Fig. 3F). First, 40 (out of 57) CT (CCC > 0.75) and 9 (out of 61) PET (CCC > 0.65) radiomic features with good robustness were selected through test/retest analysis (Table S2) and then brought into LASSO regression. With ICI groups (including both LLC and B16 models) as the training set, features significantly correlated with characteristics of CD8^+^ T cell exhaustion were identified (Table 1). Since too small amount of significant CT features were identified, each only exhibiting connection with single exhaustion characteristic, they might contribute little to construct a robust enough model. Therefore, we chose three PET features, which showed strong correlation with multiple exhaustion characteristics, to further establish a binary logistic regression model for determining the level of T cell exhaustion (median proportion of terminal Tex in the training set as cutoff). The proposed model was shown as:

**Table 1:** Radiomic features significantly correlated with characteristics of CD8^+^ T cell exhaustion selected through LASSO regression

	**CT**		**PET**	
	**Feature**	**Coefficient**	**Feature**	**Coefficient**
**Early Tex proportion among CD8**^**+**^** T cells**	SurfaceAreaDensity	0.291541	Contrast	0.003785638
			Homogeneity2	-3.455633658
			Kurtosis (Intensity Histogram)	1.696675694
			Kurtosis (Intensity Direct)	-0.482919952
**Terminal Tex proportion among CD8**^**+**^** T cells**	/	/	InverseVariance	-124.62754
**E/T**	/	/	Homogeneity2	-1.09342672
			Kurtosis (Intensity Histogram)	0.26680888
			Kurtosis (Intensity Direct)	-0.02380306
**MFI of PD-1 expression on CD8**^**+**^** T cells**	Compactness2	1069.80865	InverseVariance	-2068.013
	ConvexHullVolume3D	-32.8878		

*Tex* exhausted T cells

*E/T* the ratio of early to terminal Tex

*MFI* mean fluorescence intensity

$$T\;exhaustion\;score=-297.597\;\ast\;Inverse\;Variance(\mathrm{GLCM})+178.689\;\ast\;Homogeneity2(\mathrm{GLCM})+1.088\;\ast\;Kurtosis\;(\mathrm{Intensity}\;\mathrm{Histogram})+1.485$$

The radiomics model exhibited good discriminative ability and goodness-of-fit in both training (ICI groups) and validation (RT group) sets, with C-index of 0.818 and 0.776, Brier score of 0.156 and 0.111, respectively. The calibration curves also indicated fine conformity between the predictions and observations in terms of the risk of T cell exhaustion in both training and validation sets (Fig. S2).

Finally, the effect of the radiomics model for assessing the level of T cell exhaustion was visualized through representative pairs of PET pixel images and flow cytometric plots of RT group xenografts (Fig. 3G). Notably, GLCM-inverse variance and GLCM-homogeneity2 on PET images were found to be inversely correlated with the proportion of terminal Tex, indicating more heterogeneous local distribution of ^18^FDG uptake correlated with higher level of T cell exhaustion.

## Discussion

In this study, we described the dynamics of overall infiltration and exhausted subpopulations of tumor-infiltrating CD8^+^ T cells after ablative irradiation, and developed a PET radiomics model to differentiate T cell exhaustion status, which fitted well in both ICI and irradiation settings. We also visualized the underlying association of more heterogeneous texture on PET images with more progressed T cell exhaustion.

We assumed that when irradiation induces abundant CD8^+^ T cells to infiltrate the tumors, it might be an optimal timing to integrate ICI therapy when the less exhausted, ICI-responsive CD8^+^ T cell populations are predominant. Our study highlights an increasing overall infiltration of CD8^+^ T cells between 3 to 14 days post-irradiation followed by a sharp fall, while terminally exhausted populations significantly predominated CD8.^+^ T cells during late course of this infiltrating period, i.e. from 7 to 14 days post-irradiation. This result indicated a potential appropriate window for delivering ICI treatment between 3–7 days post-irradiation, which might reasonably explain why the overall response rate was doubled when PD-1 monoclonal antibody was given within 7 days after completion of stereotactic body radiotherapy (SBRT) in patients with metastatic NSCLC [1]

Several preclinical studies demonstrated that ablation doses of 15-25 Gy causes the massive release of antigens and death-associated molecular patterns (DAMP) ligands, and increases T-cell priming in draining lymph nodes, which activates effective antitumor immune response [34–36]. One study showed significantly increased infiltration of CD8^+^ T cells in TME on day 14 after ablative irradiation [37], which is consistent with our results. But the effect of different radiation doses on TME varies. Low-dose irradiation, e.g. 0.5-2 Gy, though insufficient to trigger anticancer response, may recruit tumor-specific CD8^+^ and CD4^+^ T lymphocytes into the tumor and reverse tumor immune desertification [38].Though only 20 Gy was investigated in our data, further studies elucidating the effect of more dose-fractionation regimens on T cell infiltration and exhaustion are necessary to help us optimize the combination strategies with ICI.

Growing evidence indicates that, in contrast to the metabolic demands of effector and memory T cells, exhausted T cells undergo metabolic insufficiency with suppressed mitochondrial respiration and glycolysis, involving restricted glucose uptake and use [24, 25]. With the understanding of distinct metabolic characteristics accompanied with T cell exhaustion, it might be a promising way to visualize the exhaustion status through ^18^FDG uptake distribution on PET images. Our findings demonstrated that local heterogeneity of ^18^FDG uptake distribution was notably correlated with progressed T cell exhaustion. It could be biologically explained that as early Tex cells developed towards terminal status, their ability of glucose uptake and use was progressively impaired, and thus, the areas where terminal Tex cells predominantly infiltrated might manifest as suppressed ^18^FDG uptake, decorating less suppressed background where early Tex cells remained dominant to form a heterogeneous landscape of ^18^FDG uptake distribution.

Recently, new radiotracers have been developed to monitor the distribution of tumor biomarkers in vivo [39] [40], and a phase I study has been conducted to perform CD8 PET imaging of TILs with a radiolabeled anti-CD8 minibody [41]. Such imaging probes might hold great potential for noninvasive PET imaging of specific biomarkers in TME and for timely evaluation of the treatment effect. However, they might be, at least by far, unable to differentiate functional subpopulations of CD8^+^ T cells since multiple-labelling probes would be more challenging in terms of safety, stability and targeting ability.

Here, we disclosed how the infiltrating CD8^+^ T cells change, as to the abundance and functionality, in ablatively irradiated TME, which might help to determine “hot” or “cold” TME as well as the optimal timing of ICI intervention. We also proposed a PET radiomics approach to noninvasively visualize the above change, based on the glucose metabolic characteristics of T cell exhaustion, which might serve as a potential solution to clinical situation when TIL biomarkers are unavailable via repeated biopsies. A noninvasive visualization tool spying the TME, cooperated with other minimally invasive, blood-based biomarkers, with the goal to define a clearer and more detailed picture of the tumor immunological parameters, will allow to real-time identify the ICI responders and optimize the combination of ICI with radiotherapy or other TME-modulating therapies [42]. However, the proposed PET radiomics approach to visualize T cell exhaustion would be further modified and verified in patients with NSCLC in future studies before applied in clinical use. And the importance of manipulating T cell exhaustion in TME should be evaluated in the clinical application of cancer immunotherapies.

## Conclusion

In summary, we described a previously uncharacterized dynamic atlas of infiltrating CD8^+^ T cell exhaustion after ablative irradiation, which indicates a potential window for ICI intervention. Basing on the glucose metabolic characteristics associated with T cell exhaustion, a noninvasive PET radiomics approach was developed to evaluate the exhaustion status, which correlates heterogeneous texture of ^18^FDG uptake distribution with ICI-resistant, progressed T cell exhaustion. This imaging predictor provides a promising solution to timely estimate immune-responsiveness of in-vivo microenvironment and the optimal timing of combined therapy; however, future validation of our findings in patients with NSCLC is needed.

## Supplementary Information

**Additional file 1: Fig S1.** Representative flow cytometry gating strategy for early-exhausted (PD-1int) and terminally-exhausted (PD-1hi) CD8+ T cells isolated from the tumor of a LLC mouse.

**Additional file 2: Fig S2.** Calibration curves of radiomics model.

**Additional file 3. Table S1.** Summary of radiomic features.

**Additional file 4.  Table S2.** Radiomics features with good robustness.

**Additional file 5.** Supplementary Methods.

## Data Availability

All data generated or analyzed during this study are included in this published article.
