# Supplementary material for: PET-based radiomics visualizes tumor-infiltrating CD8 T cell exhaustion to optimize radiotherapy/immunotherapy combination in mouse models of lung cancer
Source: Biomark Res. 2023 Jan 25;11:10. doi: 10.1186/s40364-023-00454-z (PMC9875413; doi:10.1186/s40364-023-00454-z)
Supplement: Supplementary file 1 — Additional file 1: Fig S1. Representative flow cytometry gating strategy for early-exhausted (PD-1int) and terminally-exhausted (PD-1hi) CD8+ T cells isolated from the tumor of a LLC mouse. [file 40364_2023_454_MOESM1_ESM.docx]

**Supplementary Figures**


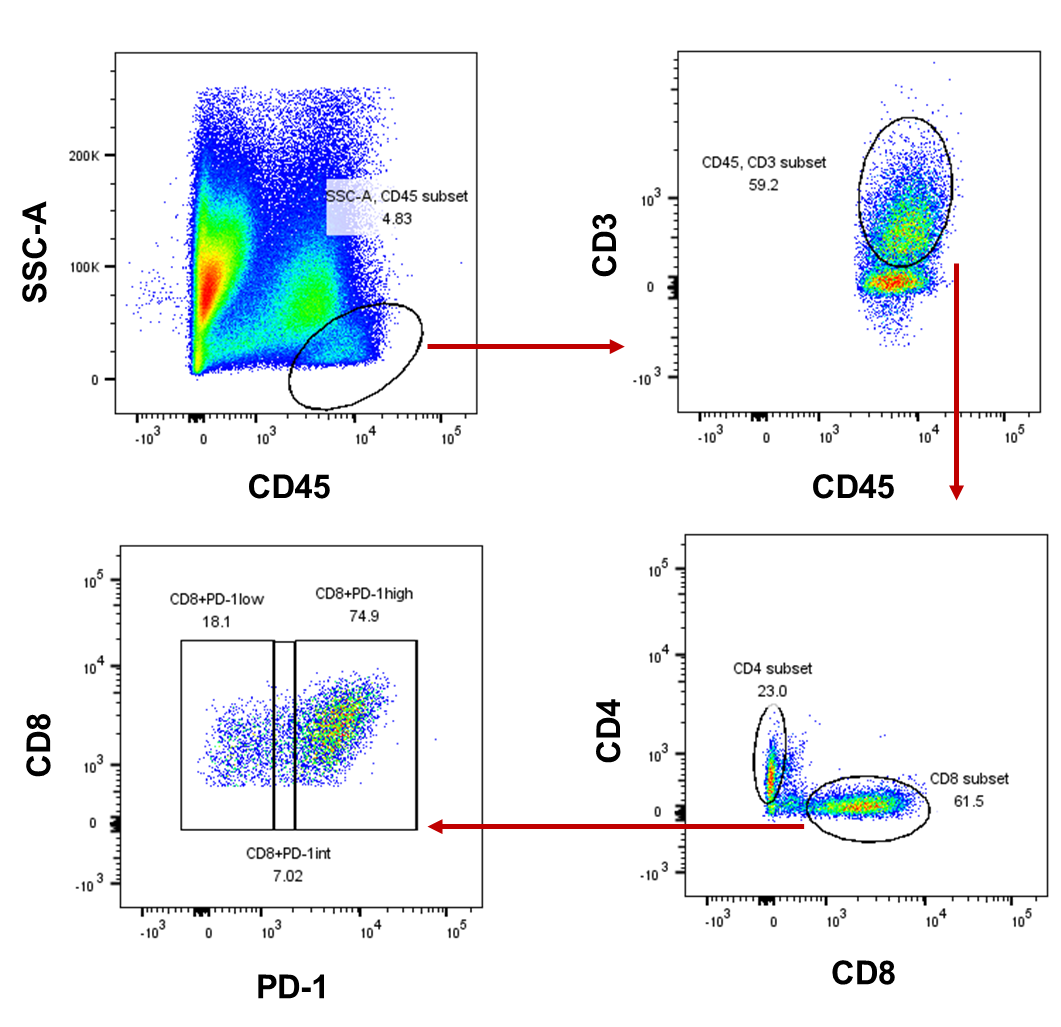


**Fig S1. Representative flow cytometry gating strategy for early-exhausted (PD-1^int^) and terminally-exhausted (PD-1^hi^) CD8+ T cells isolated from the tumor of a LLC mouse.**
