# Supplementary material for: PET-based radiomics visualizes tumor-infiltrating CD8 T cell exhaustion to optimize radiotherapy/immunotherapy combination in mouse models of lung cancer
Source: Biomark Res. 2023 Jan 25;11:10. doi: 10.1186/s40364-023-00454-z (PMC9875413; doi:10.1186/s40364-023-00454-z)
Supplement: Supplementary file 2 — Additional file 2: Fig S2. Calibration curves of radiomics model. [file 40364_2023_454_MOESM2_ESM.docx]

**
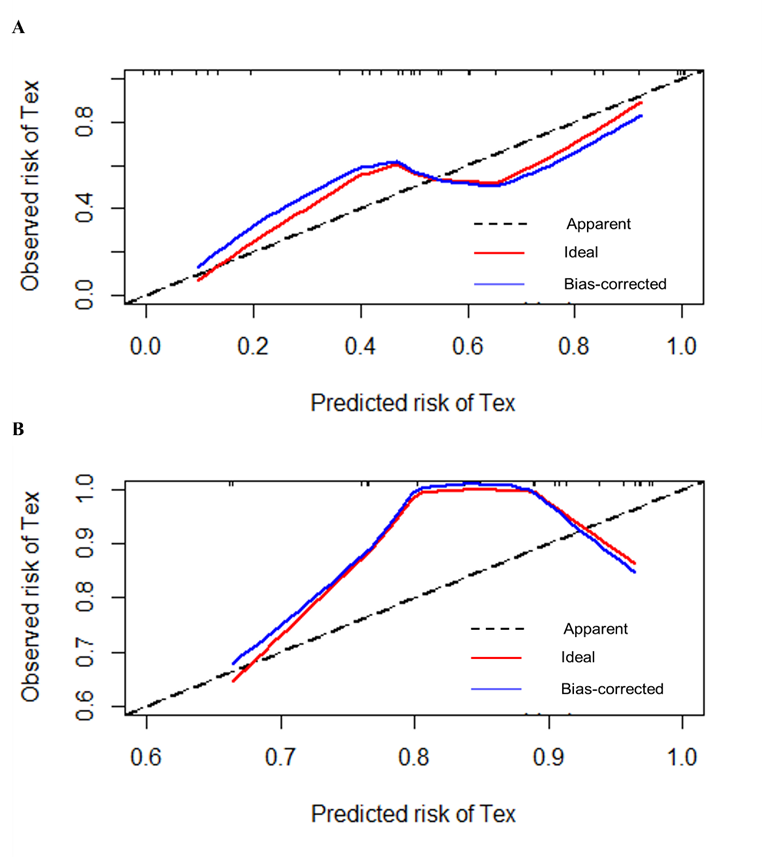
**

**Fig S2.** **Calibration curves of radiomics model.**

1. Calibration curve of ICI group (training set).
2. Calibration curve of RT group (validation set).
