# Supplementary material for: PET-based radiomics visualizes tumor-infiltrating CD8 T cell exhaustion to optimize radiotherapy/immunotherapy combination in mouse models of lung cancer
Source: Biomark Res. 2023 Jan 25;11:10. doi: 10.1186/s40364-023-00454-z (PMC9875413; doi:10.1186/s40364-023-00454-z)
Supplement: Supplementary file 3 — Additional file 3. Table S1. Summary of radiomic features. [file 40364_2023_454_MOESM3_ESM.docx]

**Table S1 Summary of radiomic features**

|  | **CT** | **PET** |
| --- | --- | --- |
| **Category** | **Features** | **Features** |
| IntensityDirect | GlobalEntropy  GlobalStd  GlobalUniformity  Energy  GlobalMax  GlobalMean  Variance | Energy  GlobalEntropy  GlobalMax  GlobalMean  GlobalStd  GlobalUniformity  Kurtosis  MeanAbsoluteDeviation  Range  Skewness  Variance |
| IntensityHistogram | Kurtosis  MeanAbsoluteDeviation  Skewness | Kurtosis  MeanAbsoluteDeviation  Skewness |
| GrayLevelCooccurenceMatrix25 | AutoCorrelation  ClusterProminence  ClusterShade  ClusterTendendcy  Contrast  Correlation  DifferenceEntropy  Dissimilarity  Energy  Entropy  Homogeneity  Homogeneity2  InformationMeasureCorr1  InformationMeasureCorr2  InverseDiffMomentNorm  InverseDiffNorm  InverseVariance  MaxProbability  SumAverage  SumEntropy  SumVariance  Variance | AutoCorrelation  ClusterProminence  ClusterShade  ClusterTendendcy  Contrast  Correlation  DifferenceEntropy  Dissimilarity  Energy  Entropy  Homogeneity  Homogeneity2  InformationMeasureCorr1  InformationMeasureCorr2  InverseDiffMomentNorm  InverseDiffNorm  InverseVariance  MaxProbability  SumAverage  SumEntropy  SumVariance  Variance |
| Shape | Compactness1  Compactness2  Convex  ConvexHullVolume  ConvexHullVolume3D  Mass  Max3DDiameter  MeanBreadth  Orientation  Roundness  SphericalDisproportion  Sphericity  SurfaceArea  SurfaceAreaDensity  Volume | Compactness1  Compactness2  Convex  ConvexHullVolume3D  Max3DDiameter  MeanBreadth  NumberOfVoxel  Orientation  Roundness  SphericalDisproportion  Sphericity  SurfaceArea  SurfaceAreaDensity  Volume |
| GrayLevelRunLengthMatrix25 | GrayLevelNonuniformity  HighGrayLevelRunEmpha  LongRunEmphasis  LongRunHighGrayLevelEmpha  LongRunLowGrayLevelEmpha  LowGrayLevelRunEmpha  RunLengthNonuniformity  RunPercentage  ShortRunEmphasis  ShortRunHighGrayLevelEmpha  ShortRunLowGrayLevelEmpha | GrayLevelNonuniformity  HighGrayLevelRunEmpha  LongRunEmphasis  LongRunHighGrayLevelEmpha  LongRunLowGrayLevelEmpha  LowGrayLevelRunEmpha  RunLengthNonuniformity  RunPercentage  ShortRunEmphasis  ShortRunHighGrayLevelEmpha  ShortRunLowGrayLevelEmpha |
