# Supplementary material for: PET-based radiomics visualizes tumor-infiltrating CD8 T cell exhaustion to optimize radiotherapy/immunotherapy combination in mouse models of lung cancer
Source: Biomark Res. 2023 Jan 25;11:10. doi: 10.1186/s40364-023-00454-z (PMC9875413; doi:10.1186/s40364-023-00454-z)
Supplement: Supplementary file 4 — Additional file 4. Table S2. Radiomics features with good robustness. [file 40364_2023_454_MOESM4_ESM.docx]

**Table S2 Radiomics features with good robustness**

|  | **CT（CCC>0.75）** | **PET（CCC>0.65）** |
| --- | --- | --- |
| **Category** | **Features** | **Features** |
| IntensityDirect | GlobalEntropy  GlobalStd  GlobalUniformity  Energy  GlobalMean  Variance | Kurtosis |
| IntensityHistogram | Kurtosis  MeanAbsoluteDeviation  Skewness | Kurtosis |
| GrayLevelCooccurenceMatrix25 | ClusterShade  ClusterTendendcy  Contrast  Correlation  DifferenceEntropy  Dissimilarity  Entropy  InformationMeasureCorr1  InformationMeasureCorr2  InverseDiffMomentNorm  InverseDiffNorm  MaxProbability  SumEntropy  Variance | Contrast  DifferenceEntropy  Dissimilarity  Homogeneity  Homogeneity2  InverseVariance |
| Shape | Compactness1  Compactness2  ConvexHullVolume  ConvexHullVolume3D  Mass  Max3DDiameter  MeanBreadth  Roundness  SphericalDisproportion  Sphericity  SurfaceArea  SurfaceAreaDensity  Volume | / |
| GrayLevelRunLengthMatrix25 | GrayLevelNonuniformity  RunLengthNonuniformity  RunPercentage  ShortRunEmphasis | LongRunEmphasis |
