# Supplementary material for: PET-based radiomics visualizes tumor-infiltrating CD8 T cell exhaustion to optimize radiotherapy/immunotherapy combination in mouse models of lung cancer
Source: Biomark Res. 2023 Jan 25;11:10. doi: 10.1186/s40364-023-00454-z (PMC9875413; doi:10.1186/s40364-023-00454-z)
Supplement: Supplementary file 5 — Additional file 5. Supplementary Methods. [file 40364_2023_454_MOESM5_ESM.docx]

**Supplementary Methods**

**Flow cytometry**

First, the tumor sample was enzymatically digested into a single cell suspension, while the spleen tissue was dissected and dissociated using a rubber pestle to acquire a single-cell suspension. Then, staining to detect cell surface antigens was performed (including Live/Dead, CD45, CD3, CD8, CD4, PD-1 staining) followed by fixation/permeabilization steps, and staining for intracellular antigens (including TIM-3, TOX and TCF-1 staining).

Gating strategy was shown as follow:

1. Aggregates were gated out using FSC-A vs. FSC-H and SSC-A vs. SSC-H and live cells were selected.
2. The leukocyte-specific marker CD45 was used to pull out the immune cell populations from the tumor cells.
3. T cells were quantified using CD3 from immune cells.
4. CD3-cells were further subdivided into CD4-cells and CD8-cells.
5. CD8-cells were then divided into PD-1^low^, PD-1^int^ and PD-1^high^ subpopulations by its low controls of CD8-cells from spleen of untreated mice (PD-1^low^), high controls of cells stained by both anti-TOX and anti-PD-1(PD-1^hi^) and between them (PD-1^int^).
